# Coming of age for COI metabarcoding of whole organism community DNA: Towards bioinformatic harmonisation

**DOI:** 10.1111/1755-0998.13502

**Published:** 2021-09-30

**Authors:** Thomas J. Creedy, Carmelo Andújar, Emmanouil Meramveliotakis, Victor Noguerales, Isaac Overcast, Anna Papadopoulou, Hélène Morlon, Alfried P. Vogler, Brent C. Emerson, Paula Arribas

**Affiliations:** ^1^ Department of Life Sciences Natural History Museum London UK; ^2^ Instituto de Productos Naturales y Agrobiología (IPNA‐CSIC) S.C. La Laguna Spain; ^3^ Department of Biological Sciences University of Cyprus Nicosia Cyprus; ^4^ Institut de Biologie de l’ENS (IBENS), Département de Biologie École Normale Supérieur, CNRS, INSERM, Université PSL Paris France; ^5^ Department of Life Sciences Imperial College London Silwood Park Campus Ascot UK

**Keywords:** animal communities, bioinformatics, COI barcode, community ecology, high‐throughput sequencing, metabarcoding

## Abstract

Metabarcoding of DNA extracted from community samples of whole organisms (whole organism community DNA, wocDNA) is increasingly being applied to terrestrial, marine and freshwater metazoan communities to provide rapid, accurate and high resolution data for novel molecular ecology research. The growth of this field has been accompanied by considerable development that builds on microbial metabarcoding methods to develop appropriate and efficient sampling and laboratory protocols for whole organism metazoan communities. However, considerably less attention has focused on ensuring bioinformatic methods are adapted and applied comprehensively in wocDNA metabarcoding. In this study we examined over 600 papers and identified 111 studies that performed COI metabarcoding of wocDNA. We then systematically reviewed the bioinformatic methods employed by these papers to identify the state‐of‐the‐art. Our results show that the increasing use of wocDNA COI metabarcoding for metazoan diversity is characterised by a clear absence of bioinformatic harmonisation, and the temporal trends show little change in this situation. The reviewed literature showed (i) high heterogeneity across pipelines, tasks and tools used, (ii) limited or no adaptation of bioinformatic procedures to the nature of the COI fragment, and (iii) a worrying underreporting of tasks, software and parameters. Based upon these findings we propose a set of recommendations that we think the metabarcoding community should consider to ensure that bioinformatic methods are appropriate, comprehensive and comparable. We believe that adhering to these recommendations will improve the long‐term integrative potential of wocDNA COI metabarcoding for biodiversity science.

## 1 INTRODUCTION

Metabarcoding of DNA extracted from community samples of whole organisms (whole organism community DNA, wocDNA) is a reliable and cost‐efficient tool to study the biodiversity of metazoan communities (Bush et al., 2019; Ji et al., 2013; Porter & Hajibabaei, 2018). This approach, which has also been referred to as community DNA (e.g., Andújar et al., 2018; Deiner et al., 2017) or bulk sample DNA (e.g., Braukmann et al., 2019; Yu et al., 2012) metabarcoding, primarily differs from other approaches such as eDNA (environmental or extra‐organismal DNA; Taberlet et al., 2012) or iDNA (vertebrate DNA ingested by invertebrates; Schnell et al., 2012) in that the source material is a community of whole organisms collected through direct trapping or collection (e.g., malaise traps; Ji et al., 2013, or canopy fogging; Creedy et al., 2019) or separated from an environmental sample (e.g., from soil; Arribas et al., 2016, or water; Suter et al., 2021). As a consequence, compared with eDNA and iDNA, wocDNA samples are characterised by (i) a comparatively low level of DNA degradation in the target species, (ii) a low proportion of nontarget species, and (iii) the possibility for complementing, refining and/or validating metabarcoding‐derived community data against other conventional morphological and molecular methods.

Metabarcoding of wocDNA samples is increasingly employed in community ecology, evolutionary ecology, biogeography, conservation biology, environmental management, and policy and decision‐making (e.g., Bush et al., 2020; deWaard et al., 2019; Leese et al., 2018). Metazoan wocDNA metabarcoding has been adapted from pioneering approaches developed to inventory and characterise microbial diversity (e.g., Gilbert et al., 2010; Sogin et al., 2006). The majority of these adaptations have focused on sampling, and molecular laboratory steps, including adapted protocols to (i) sample, separate, enrich and/or clean animal wocDNA samples (Creedy et al., 2019; Fonseca et al., 2010, 2011), (ii) perform wocDNA extractions (Marquina et al., 2019; Nielsen et al., 2019), (iii) design and evaluate primers (Braukmann et al., 2019; Elbrecht et al., 2019; Elbrecht & Leese, 2017), optimise amplification (Krehenwinkel et al., 2017) and prepare libraries (Yang et al., 2021). There is a growing consensus on the use of the mitochondrial cytochrome oxidase subunit I (COI) barcode, rather than other markers widely used for metabarcoding of nonmetazoan communities, as the standard for wocDNA metabarcoding due to the range of COI primers with demonstrated efficiency (Braukmann et al., 2019; Elbrecht & Leese, 2017), and the potential of COI to improve the utility, resolution and reliability of wocDNA metabarcoding data (Andújar, Arribas, Gray, et al., 2018; Turon et al., 2020).

However, in contrast to these advances in sampling and molecular processing, there has been limited effort to review and evaluate how bioinformatic processing has been adapted to metazoan wocDNA samples and the COI barcode, nor to examine consistency in bioinformatic approaches across the field. Broadly, bioinformatic tasks involve the computational cleaning, filtering and analysis of raw sequence data to produce biodiversity data comprising taxonomic units and their incidence across samples, implemented in a particular order (a “pipeline”). There are a wide array of software tools available for performing different bioinformatic tasks, from standalone tools to catch‐all software packages (e.g., OBItools Boyer et al., 2016; QIIME Caporaso et al., 2010; USEARCH/UPARSE Edgar, 2013; and its open‐source derivative VSEARCH Rognes et al., 2016). These tools have largely been developed for metabarcode loci other than the COI region, with very few tools explicitly developed for protein coding metabarcodes (although see Andújar et al., 2021; Nugent et al., 2020; Ramirez‐Gonzalez et al., 2013). To fully capitalise on the COI barcode for metabarcoding, bioinformatics should be specifically tailored to its evolutionary properties, such as the ability to interrogate the amino acid translation, and accounting for established patterns of sequence variation in protein coding genes for strict filtering. Additionally, metabarcoding employs a number of key bioinformatic tasks for which multiple alternative algorithms have been developed (e.g., denoising algorithms), with considerable variation in outcomes depending on parameters and thresholds applied.

The structure of a bioinformatic metabarcoding pipeline will depend strongly on the research aim, amplification and sequencing protocols, target locus, and target biodiversity fraction. The diversity of bioinformatic tasks and the software approaches to implement them is of course beneficial for designing appropriate pipelines, but such heterogeneity may also restrict integrated, standardised and synergistic growth in the field. As metazoan wocDNA metabarcoding becomes more accessible to researchers from a range of fields and backgrounds, harmonisation of bioinformatic approaches is important to ensure (i) high‐quality, reproducible data amenable to qualitative or quantitative reviews and meta‐analysis across studies, and (ii) a reliable, consistent methodology for wider implementation, development and expansion of wocDNA metabarcoding. We consider harmonisation not to mean strict prescription of the tasks and software to use, nor their order. Instead a harmonised field would recognise the diversity of approaches available, while recording key steps and establishing the effects of parameter choice on the outcome of metabarcoding studies. This approach could be enabled by the adoption of universal aligned standards for data generation and processing, while allowing for flexibility in implementation to adapt to varying research goals and take advantage of novel methodological development.

Harmonisation requires comprehensive examination of current practice to understand the aims and approaches of prior work, and a synthesis of the successes and failures in past implementations for the purposes of elaborating a framework to guide future research. Therefore it is our aim to summarise the state of the art for bioinformatic processing of metazoan wocDNA COI metabarcoding, and in doing so assess the potential for harmonisation. To this end, we performed a systematic review of peer‐reviewed studies, collating information on the different bioinformatic pipelines, tasks and tools used in wocDNA COI metabarcoding in >100 recent studies (2011–2020). We use this data to (i) describe the diversity, heterogeneity and reproducibility of the bioinformatic procedures followed, (ii) identify the extent to which these procedures are compatible with the evolutionary properties of the COI marker, and (iii) identify the key bioinformatic tasks, provide a framework for successful metabarcoding bioinformatics and make recommendations towards harmonised bioinformatic procedures for metazoan wocDNA COI metabarcoding.

## 2 MATERIALS AND METHODS

### 2.1 Bibliographic search and screening

We focused this work on studies using whole organism community DNA (wocDNA) metabarcoding. In general, we define wocDNA samples as those where the target organisms were: (i) probably alive at the time of sampling, (ii) present as a largely complete specimen, and (iii) potentially identifiable using classical methods of morphological analysis. We exclude eDNA and iDNA metabarcoding due to the potentially different bioinformatic processing needs associated with these samples. In particular, eDNA and iDNA bioinformatic methods need to accommodate degraded DNA and a potentially high proportion of nontarget reads. Furthermore, in many cases wocDNA metabarcoding is directly comparable to direct observation of specimens and conventional methods of taxonomic assignment not available for eDNA metabarcoding (Ji et al., 2013; Aylagas et al., 2016). This allows for more objective stringency thresholds in bioinformatic filtering and delimitation of operational taxonomic units (OTU).

We conducted a systematic search of peer‐reviewed studies in the Web of Science (WOS) Core Collection (Science Citation Index Expanded, 1900–present) on 3 November 2020, using the search “TS = (metabarcoding) NOT TS = (*micro* OR *bacteria* OR *myco* OR *archaea* OR fungi OR plant OR eDNA OR environmental DNA)”. These search parameters were selected in order to obtain a comprehensive set of wocDNA metabarcoding studies limited to Metazoa.

The systematic search resulted in 692 records, which were screened to to select only those studies that: (i) amplified some portion of the standard COI barcode “Folmer” region (Folmer et al., 1994), (ii) fit our definition of wocDNA samples, comprising mixtures of organisms extracted from the substrate, and (iii) provided a characterisation of metazoan communities. Studies targeting extra‐organismal DNA (i.e., eDNA, iDNA) were excluded. We included studies of experimental mock communities composed of mixtures of DNA extracted from individual specimens or mixtures of specimens, and we also included studies where the target organisms remained partially or completely within an environmental substrate upon which DNA extraction was performed (e.g., parasites within a host, arthropods within soil), if the principal target was the whole organism community DNA. After reviewing the final set of filtered papers, 24 additional papers fitting the selection criteria but not present in the systematic WOS search were also included. A total of 111 articles constituted the set of core papers for subsequent assessment (see Table S2 for a complete list).

### 2.2 The core studies

All papers were systematically processed to record (i) the research aim and type of samples analysed, (ii) the bioinformatic tasks and pipelines implemented, and (iii) the software tools used and the reproducibility of the bioinformatic procedures employed. We define these terms as follows:

● Task: a specific, self‐contained action in a pipeline, generally with a clearly‐defined purpose and performed by a single tool. e.g., demultiplexing.
● (Software) tool: a specific piece of software, or a specific identifiable function within a software package. e.g., Cutadapt, or USEARCH cluster_otus.
● Pipeline: a sequence of steps in a specific order, each step performing a particular task and using a specific tool.

The research aim was categorised according to whether the focus was (i) the comparison of molecular and/or bioinformatic procedures for metabarcoding, (ii) a proof‐of‐concept or feasibility study into the success of metabarcoding for uncovering accurate community data in the taxon/community/biome studied, or (iii) principally the study of ecological patterns and processes. We recorded whether the metabarcoded communities were sampled from marine, freshwater, terrestrial biomes or from a host species, and finally if the targets were invertebrates or vertebrates.

Subsequently, the bioinformatic procedures for each paper were systematically parsed to identify the different tasks implemented. A total of 30 distinct bioinformatic tasks were identified starting from initial procedures on raw sequencing files through to the generation of community tables (see Table 1 for a description of each task). We focused solely on bioinformatic tasks that were presented as necessary for the generation of information about the occurrence or incidence of taxonomic units in the sampled communities (i.e., community data), and the taxonomic identity of these units. For example, we did not record any steps performing phylogenetics with a final OTU set, although we recorded steps where phylogeny‐based methods were used as part of OTU delimitation and filtering. Similarly, we recorded tasks that performed filtering of community data for the purposes of removing OTUs or OTU records arising from erroneous sequences or from cross‐talk/contamination (Edgar, 2018), but we did not record tasks that filtered community data for the purposes of statistical correction, such as normalisation or rarefaction.

**TABLE 1:** Table of all bioinformatic tasks performed across the core papers set

Task group	Task	Description	Number of papers reporting task	Number of papers not reporting software	Total number of software tools	Total number of software functions	Number of papers performing manually
Read preparation	Quality control	*Generating a report of sequence quality information from a sample or set of samples ‐ no modification is done to data*	19	0	4	4	0
	Adapter trimming	*Trimming of sequencing adapters*	9	1	6	6	0
	Demultiplexing	*Separation of sequences from a mixed pool into separate pools based on the occurrence of a unique set of bases (index or tag)*	55	17	16	19	0
	Pair merging	*The assembly of mate pair reads into a single contig*	63	1	10	18	0
	Quality trimming	*The removal of bases from either or both ends of sequences in a pool based on quality scores*	20	1	8	10	0
	Mate pairing	*The identification and syncronisation of mate pair reads between two samples, often involving arranging reads in identical orders and/or removal of reads without a mate pair*	3	0	3	3	0
	Primer trimming	*Trimming of PCR primers*	66	8	15	17	0
	Reverse complementation	*Reverse complementing the sequences in a pool*	7	3	2	2	0
	Sequence conversion	*Converting sequences from fastq to fasta*	3	0	2	3	0
	Length trimming	*The removal of bases from either or both ends of sequences in a pool, either the removal of a fixed number of bases or the removal of a variable number of bases to reduce sequences to a standard length*	10	3	6	7	0
	Pair concatenation	*Concatenating mate pair reads into a single contig (where reads don't overlap)*	8	4	4	4	0
	Assembly	*The assembly of reads into contigs, applied when more than one pair of overlapping fragments have been metabarcoded*	6	0	4	4	0
	Degapping	*Removal of gaps from sequences*	1	0	1	1	0
Sequence processing	Dereplication	*The removal of duplicate reads to retain only unique sequences in a pool; often the total number of copies of a sequence is recorded in the header of the retained sequence*	58	10	11	19	0
	Size sorting	*The sorting of a fasta file according to a size annotation in the header*	10	2	3	4	0
Filtering	Quality filtering	*Removal and*/*or trimming of sequences from a pool based on quality information*. *Also often converts from fastq to fasta*.	81	11	20	27	0
	Similarity filtering	*Removal of sequences based on similarity to an alignment, either based on sequence identity or alignment position*	9	1	4	4	0
	Length filtering	*The removal of sequences from a pool that are less than, more than, or fall within or outside of a specified length threshold or thresholds*	54	21	17	23	0
	Preclustering	*Reduction of sequence variation in a dataset prior to further processing ‐ a form of denoising*	12	1	3	6	0
	Denoising	*The removal of reads containing putative PCR or sequencing errors based on statistical assessment*	18	1	8	8	0
	Normalisation	*A process by which the number of sequences for each of a set of samples is reduced where necessary such that the output set of samples all have the same number of sequences while maintaining the relative frequencies of OTUs*	2	0	1	1	1
	Chimera filtering	*The filtering of putative chimeric assemblies from a pool of mate paired reads*	63	4	6	16	1
	Translation filtering	*Removal of sequences from a set of sequence based on their translation, usually removing sequences with inframe stop codons or frameshifts due to erroneous indels or substitutions caused by sequencing errors*	22	3	11	12	0
	Frequency filtering	*Removal of sequences based on their frequency in a pool*	51	37	11	15	1
	Taxonomy filtering	*Removal of sequences based on an assigned taxonomy or a taxonomic classification*	9	5	1	1	1
	Mistag filtering	*Removal of sequences based on putative tagging errors*	3	1	1	1	0
Data generation	OTU delimitation	*The grouping of a set of sequences into OTUs by some method*	84	5	12	22	0
	OTU mapping	*The mapping of sequences to OTUs to provide read counts for each OTU*	30	3	7	11	0
	Uncurated taxonomic assignment	*The assignment (identification or classification) of taxonomy to OTUs using a global uncorated reference database (e.g., GenBank, BOLD)*	55	2	11	13	0
	Reference taxonomic assignment	*The assignment (identification or classification) of taxonomy to OTUs using a purpose‐built and/or specially curated reference set of sequences*	60	9	18	23	1

Note

Tasks are grouped into four groups by broad purpose, and a detailed definition of each task is given along with summary statistics of the implementation of each task across the 111 papers. For a list of the software used for each task, Table S1 is an expanded version of this table.

Once the different tasks implemented by each article were identified, the pipeline used was also recorded based on the order in which the different tasks were mentioned in the text, figures, Appendix S1 and/or cited papers. Where multiple mutually exclusive tasks were employed for the purposes of comparison of pipelines, we recorded that pipeline that the authors concluded to be empirically superior, or from which the authors used the output data for subsequent analysis. A detailed description of the systematic processing of methods is described in the Appendix S1.

For each of the bioinformatic tasks identified across the papers, we calculated (i) the number of papers implementing the task, (ii) the task's relative position within the pipelines, (iii) the information reported on the software, version and parameters used, and (iv) the homogeneity in the software tools used to implement the task. We assessed homogeneity by calculating two indices, the software homogeneity rate and the software dominance rate. Software homogeneity rate for a given task (*t*) in a given year (*y*) was calculated as:

$$1‐\frac{S_{\mathrm{yt}}}{P_{\mathrm{yt}}}$$

where *s* is the number of different software tools used and *p* is the number of papers. The software dominance rate was similarly calculated as:

$$\frac{n}{P_{\mathrm{yt}}}$$

where *n* is the number of papers for a given task in a given year that used the most common software tool for that task in that year. Finally, we also summarised temporal trends in both the reporting and software heterogeneity of each task.

## 3 RESULTS AND DISCUSSION

### 3.1 Diversity of bioinformatic methods

**FIGURE 1:**
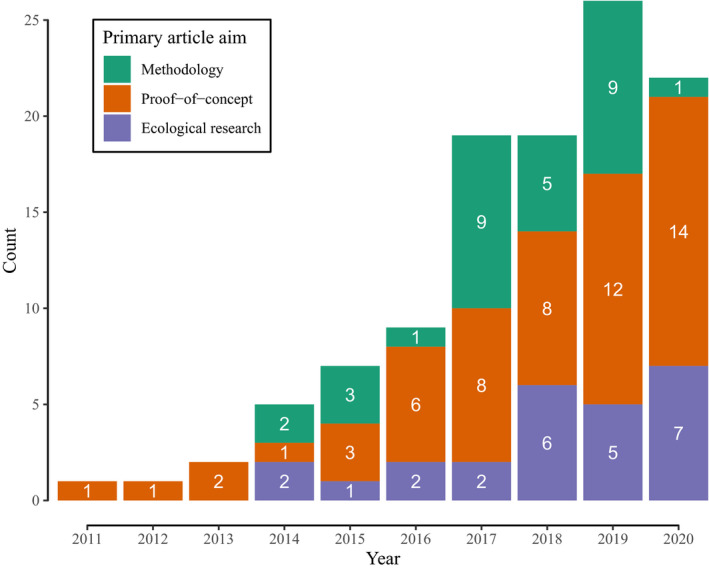
Year of publication of the articles in the core papers set. Bar fills and numbers refer to the number of articles within each research aim category. Note that only articles indexed by Web of Science by 3rd November 2020 were included

The 111 selected papers were published in 36 different journals with a broad focus on ecology and molecular ecology. There has been a steady increase in the number of papers published in this domain over time (Figure 1). The earliest year of publication was 2011, but 77% of all papers were published in the last 4 years (2017–2020, *n* = 86, Figure 1). Almost all papers studied invertebrate communities (*n* = 108). Forty‐five papers were focussed on terrestrial communities, 31 on freshwater, 30 on marine and five on parasite communities collected from a host vertebrate (see Table S2 for all the details on the core papers set).

Despite a clear trend for increased use of wocDNA COI metabarcoding, the field remains in a relatively early stage of implementation, reflected in the fact that in half of all papers (*n* = 56, *n* = 38 in the last 4 years) metabarcoding was undertaken as a proof‐of‐concept and the authors primarily discussed the feasibility of this method for the studied ecological system. Only 25 papers considered the sample sizes and metabarcoding procedures sufficiently rigorous to answer ecological questions. Thirty papers were primarily methodological, assessing the influences of primer choice, laboratory protocols and/or sequencing methods. However, within the methodological category, no paper solely studied the effect of bioinformatic pipeline choices. Indeed, only eight out of the 111 papers clearly stated that they compared different tools for the same task, despite the use of 116 software tools (i.e., discrete pieces of software or functions within software packages) in our final count. These results illustrate the timely nature of this review, highlighting the inconsistent implementation of bioinformatic methods, in contrast to the relative maturity and harmonisation of field and laboratory methodologies.

### 3.2 High heterogeneity in tasks and pipelines

**FIGURE 2:**
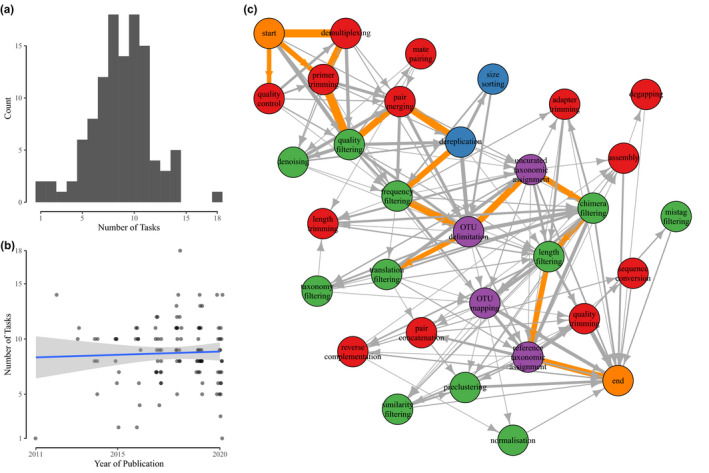
Bioinformatic pipelines implemented by the core papers set. (a) Frequency distribution of the number of tasks by study, (b) Number of tasks by study against the year of publication, with best fit regression line in blue with shaded 95% confidence intervals around the line. Slight horizontal jitter added to points to better show density. (c) Network diagram of tasks and different pipeline routes through these tasks. All pipelines start and end on the respective orange nodes. All other nodes are coloured according to the four main categories of bioinformatic tasks; red for read preparation tasks, blue for sequence processing, green for filtering and purple for data generation tasks. Arrows link tasks performed consecutively, with direction of arrow showing order of tasks. Thickness of arrows shows relative frequency of pairs of consecutive tasks. Arrows coloured orange are the top 10% of consecutive task pairs by relative frequency; note that while this illustrates a possible complete pipeline from Start to End, this “average” pipeline is not in fact performed by any of the papers assessed by this review

**FIGURE 3:**
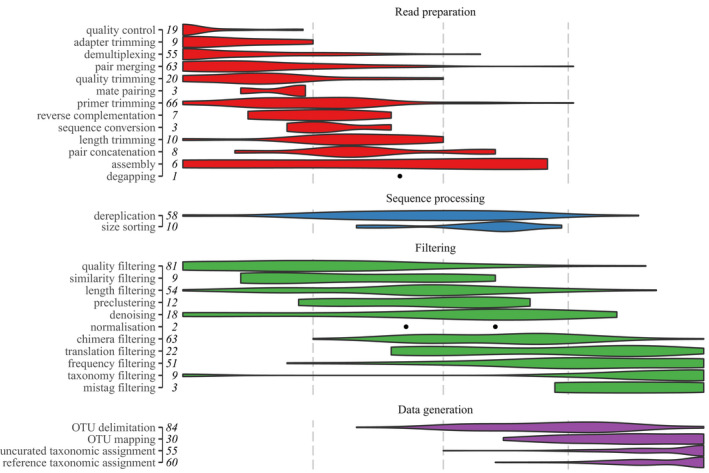
Violin plot of standardised task position within pipelines. Increasing x‐axis position denotes later placement of task within pipelines, vertical dashed lines denote 25%, 50% and 75% of the way through the pipeline, respectively. Tasks are separated into task groups and ordered within task group by mean standardised pipeline position. Points denote task positions where tasks occurred too infrequently to compute density profile for violin plots. Values report the total number of papers implementing each task

The variety of bioinformatic pipelines reported across the 111 papers employed 108 unique pipelines, that is, sets of bioinformatic tasks carried out in a specific sequence. Three pipelines were used twice; in two of these cases, a group of authors replicated their pipeline exactly, in the other case the pipeline as reported consisted solely of a single step of searching raw reads against a reference set. Although some of these pipelines were similar, with minor modifications to the order, or the addition/removal of a few tasks, the heterogeneity of pipelines is remarkable. There was also high heterogeneity in the number of tasks implemented within each pipeline, ranging between 1 and 18 tasks, with half of the articles reporting fewer than nine distinct bioinformatic tasks (Figure 2a). There was no particular trend in the number of tasks implemented over time (Figure 2b). The order in which these tasks were implemented also differed greatly (Figure 2c), although there was a tendency for certain tasks to be performed within similar general stages within pipelines, that is, read preparation‐based tasks tend to be implemented at the initial steps of the pipelines, followed by filtering‐based tasks and data generation tasks (Figure 3).

Heterogeneity in the sequence of tasks may reflect the careful design and adaptation of bioinformatic procedures within each study to the type and structure of sample and sequence data and/or the specific research question, rather than the simple duplication of previously published pipelines. However, high heterogeneity may equally result from the omission of important tasks or their inappropriate implementation within the pipelines, and so result in low comparability, integration and replication across studies. One clear example of this is associated with the Filtering tasks of removal of erroneous sequence reads. Denoising (i.e., the removal of sequencing errors based on models of error frequency parameterised by between‐sequence similarity, error sensitivity and/or relative frequency), was employed in just 18 studies and its relative position within the pipelines was highly variable (see Table 1 and Figure 3). While some sequencing errors will be disregarded during OTU clustering, failure to incorporate denoising can lead to false OTUs and thus OTU inflation (Shum & Palumbi, 2021). While in metabarcoding literature denoising and OTU clustering are seen as mutually exclusive procedures, the high intraspecific diversity of COI means that employing both tasks in a complementary and comparative framework can be extremely informative, and it is in fact a novel promising area for COI metabarcoding (see e.g., Antich et al., 2021; Arribas, Andújar, Bidartondo, et al., 2021; Arribas et al., 2021; Brandt et al., 2021). Furthermore, the trend towards examining haplotypic variation in metazoan wocDNA metabarcoding through use of amplicon sequence variants (ASVs, Callahan et al., 2017) requires minimising the number of spurious sequences, relying on stringent filtering such as denoising. Similarly, filtering to remove sequences with low copy number (that are often considered highly likely to be erroneous) was reported in only half (*n* = 57) of the studies, despite being generally recommended (Calderón‐Sanou et al., 2020; Ficetola et al., 2017) and a critical step for reducing spurious sequences surviving denoising including nuclear mitochondrial (NUMT, Lopez et al., 1994) copies (Andújar et al., 2021). It should be noted that while many task absences are cases of underimplementation, some may also be underreporting (see below).

### 3.3 Infrequent adaptation of pipelines to COI

The COI locus differs from many other metabarcoding loci (e.g., 18S, 16S, 12S, ITS) in that it is a protein coding gene, imparting strict expectations of amplicon sequence read properties that can be exploited in metabarcoding bioinformatics (Andújar, Arribas, Yu, et al., 2018). However, the adaptation of pipelines to this fragment are in general rarely implemented in the papers of the core set. For example, only 22 papers (20%) used amino acid translations to identify erroneous sequences (“translation filtering”), using 11 different software tools for the task. The reason for low implementation of translation filtering is probably that none of the major metabarcoding software packages include functions for translation filtering and that the available methods are limited. Those papers that carry out translation filtering do so by using one of three main approaches: (i) sequences are viewed and translated in a GUI application such as Geneious (https://www.geneious.com) or MEGA (Kumar et al., 2018), and those with stop codons manually removed, (ii) sequences are processed through a custom script, some of which are available on github but none of which are used by research groups separate from the author, and (iii) sequences are aligned against references using MACSE (Ranwez et al., 2011) and those containing indels or stop codons are removed. The first option is time consuming and prone to human error, and custom scripts are challenging to document and maintain for a wider number of users. While MACSE is the most frequent single approach, it is computationally efficient only for small data sets. There may be some potential in the recent coil R package (Nugent et al., 2020) that uses Hidden Markov Models to identify and filter translation‐based errors and appears to scale well to large data sets, although the R implementation presents a slight barrier to efficient inclusion in pipelines. Furthermore, the majority of translation filtering approaches are based solely on removing stop codons, while there may be other potential avenues for filtering based on amino acid translation. The extent to which expectations for protein structural properties can be applied to metabarcoding sequences for filtering other nonsynonymous errors has been poorly explored (but see Antich et al., 2021; Turon et al., 2020). It should be further noted that studies covering a wide range of metazoa and wishing to employ translation filtering may need to employ multiple different translation tables, which would require sequences to be taxonomically sorted prior to this step and then filtered separately, adding further complexity and potential for error.

In addition to the potential of amino acid translation, the protein coding nature of COI leads to relatively stricter expectations of amplicon length. However, only half (*n* = 54) of papers reported using length filtering, despite this being a relatively trivial procedure and with functions available in all metabarcoding software packages and as options in many more software tools. There may be some underreporting here; given the implementation of a length filtering parameter in many software tools that have a different primary purpose, authors may not have explicitly reported that length thresholds had been applied as part of a different procedure (note that we recorded when a single tool was reported to have fulfilled multiple tasks). Despite length filtering being widely available, and the relative algorithmic simplicity of implementation, there are no length filtering tools that allow for specification of thresholds outside of a simple minimum‐maximum range, despite the internal barcode region of protein coding genes generally being expected to vary in length only by multiples of three bases. While trivial to implement this programmatically for an experienced bioinformatician, this lack of straightforward user‐friendly availability presents a barrier to appropriate threshold implementation by those with less experience.

### 3.4 Severe underreporting and increasing heterogeneity in the tools used for bioinformatic tasks

Of the 30 bioinformatic tasks identified (see Table 1 for a description of the tasks), only 11 were implemented in more than half of the papers (*n* < 55) (Figure 3). Quality filtering (*n* = 92) and OTU delimitation (*n* = 89) were the tasks most reported. Some of the less reported tasks were those associated with uncommon bioinformatic requirements of metabarcoding data, such as assembly or degapping; others have become redundant with modern computational power, such as preclustering. Low reporting of such tasks is probably an accurate reflection of rare implementation; however, there are many other tasks that are fundamental in metabarcoding bioinformatics but are poorly reported. For example, primer trimming was only reported by just over half of the papers (*n* = 67), yet is a completely necessary step. Similarly, adapter trimming was underreported (*n* = 21); while it is likely that in the majority of cases this is implemented by sequencing facilities prior to the authors receiving data, its reporting, including parameters and tools used, is fundamental to verify stringency of the read preparation procedures. The mapping of by‐sample reads to OTUs was reported by only one third (*n* = 30) of the papers that employed OTU delimitation, despite this being a necessary step for the production of ecological data for downstream analysis. Furthermore, OTU mapping is not a trivial step; the level of filtering/processing performed on the reads used for mapping (as opposed to filtering/processing performed on the sequences used for OTU delimitation), and the similarity threshold and tie‐breaking algorithm employed to assign reads to OTU clusters could all substantially affect the community data generated. The accurate reporting of this step is important to assess the validity of a pipeline, its comparability across studies, and/or its ability to be reproduced.

**FIGURE 4:**
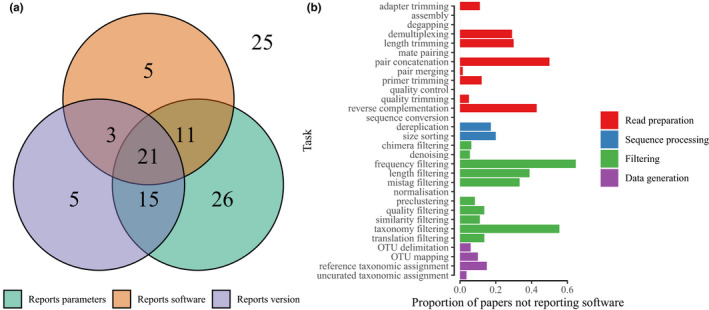
Plots summarising the reporting of three key aspects of bioinformatic tools (software name, version and parameters) by the core papers. (a). Venn diagram shows the number of papers fully reporting each detail, that is, giving the software used for every task reported, and giving the parameters and version for each task where software is given; 86 papers reported at least one of the three details for all steps, 25 further papers failed to fully report all three details in all steps. (b) Bar chart details the proportion of papers employing a specific task that failed to report the software used for that task, with longer bars denoting a greater proportion of papers not reporting software for that specific task

**FIGURE 5:**
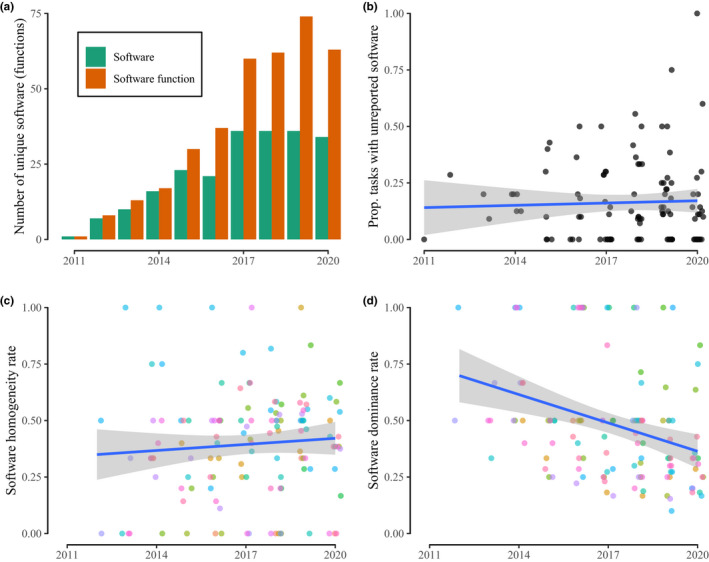
Consistency in software reporting and use over time. (a) The total number of unique software functions reported across all papers for each year of publication. (b) For each paper, the proportion of the total number of bioinformatic tasks for which the software used for a task was not reported. (c) The software homogeneity rate, calculated only when more than one paper reported a task in a given year. A value of 1 means all papers used the same tool for a given task in a given year. (d) The software dominance rate, calculated only when more than one paper reported a task in a given year. A value of 1 means all papers used the same tool for a given task in a given year. (b–d) Best fit regression lines are shown in blue with shaded 95% confidence intervals around the line. Horizontal jitter added to points to illustrate density within years; (c and d) colours denote different tasks, see Figure S1

In addition to the clear underreporting of tasks within the pipelines as discussed above, the reporting of the bioinformatic tools and parameters used for those tasks cited in the papers was also very poor (Table 1). Only 21 of the 111 studies reported software name, version and parameters used for all of the bioinformatic tasks implemented, and 25 failed on all three counts (Figure 4a). When considering the degree of underreporting by task (Figure 4b), the most underreported software were used for some of the most perfunctory tasks (e.g., frequency filtering, length filtering, dereplication) that can be easily reproduced using many equivalent tools. Nonetheless, there remains relatively widespread underreporting, and this has remained unchanged over time (Figure 5b).

Within the reported software, we identified 93 independent pieces of software used in metabarcoding bioinformatic pipelines (Table S3), of which 27% (25) were software packages. When taking into account distinct functions within packages, a total of 169 unique software tools were recorded; however, this is probably an inaccurate picture given low reporting rates of functions used within software packages. There is a clear increase in the number of different software and software functions employed across all papers over time (Figure 5a). Examining the diversity of software used within tasks over time, controlling for the number of papers published, there is limited improvement in homogeneity and a decrease in dominance of software (Figure 5c,d). Given that the number of metabarcoding publications is increasing year‐on‐year, there is thus a concomitant increase in the diversity of software used for a given task, and previously commonly used software are being used less (Figure 5c,d). These trends reflect that while new software tools are constantly being made available for metabarcoding, uptake is not consistent across the field and while some researchers use more recent tools, many researchers continue to use older methods, diversifying the field.

### 3.5 Toward a bioinformatic harmonisation of COI metabarcoding for metazoan wocDNA samples

Our results show that the increasing use of wocDNA COI metabarcoding for metazoan diversity is characterised by a clear absence of bioinformatic harmonisation, and the temporal trends show little change in this situation. The reviewed literature showed (i) high heterogeneity across pipelines, tasks and tools used, (ii) limited or no adaptation of bioinformatic procedures to the nature of the COI fragment, and (iii) a worrying underreporting of tasks, software and parameters.

The development of metabarcoding as a method for community ecology began with microbial studies over a decade ago, which have revealed the extensive diversity of bacteria and archaea on our planet and demonstrated the potential of metabarcoding for global biodiversity syntheses (Bates et al., 2013; Thompson et al., 2017). Although the integration and meta‐analysis of microbial community data from independent studies is still challenging (e.g., Ramirez‐Gonzalez et al., 2013), the success of international consortia such as the Earth Microbiome Project (EMP, Gilbert et al., 2010, 2014) has promoted the development of a harmonised framework for data generation and analyses within microbial eDNA research (see e.g., Tedersoo et al., 2015).

Through the adaptation of the microbial metabarcoding method to wocDNA samples, specific protocols to sample, sort and enrich community samples for wocDNA metabarcoding have been developed, targeting different taxonomic fractions and types of samples (e.g., Andújar, Arribas, Gray, et al., 2018; Arribas et al., 2016; Creedy et al., 2019; Elbrecht & Leese, 2017; Fonseca et al., 2010; Yu et al., 2012). Additionally, recent efforts to adapt and optimise existing methods are increasing efficiency and versatility, for example through nondestructive DNA extraction techniques that retain specimens for morphological vouchering (Marquina et al., 2019; Nielsen et al., 2019), or library preparation techniques tailored to metazoan samples (Yang et al., 2021). Although wocDNA COI metabarcoding remains in an expansive phase of development, standardisation in field and laboratory methods are emerging. This is in part boosted by collaborative initiatives such as the BIOSCAN initiative and its regional extensions (e.g., BIOALPHA), the Kruger Malaise Program, SITE‐100, the Insect Biome Atlas Project, LIFEPLAN, and iBioGen (Arribas, Andújar, Bidartondo, et al., 2021; Arribas, Andújar, Salces‐Castellano, et al., 2021).

In contrast, there has been little advance in the development and validation of best practices associated with the bioinformatic processing of wocDNA COI metabarcoding data (but see Yang et al., 2021 for error reduction). Outside of taxonomic assignment tasks for which adaptations and parameterization for using the COI barcode fragment have been further discussed (see e.g., Hleap et al., 2021), the discussion of customising or parameterising tools for the purposes of working with wocDNA COI metabarcoding is very rare (but see e.g., Andújar, Arribas, Gray, et al., 2018; Andújar et al., 2021; Antich et al., 2021), with most papers simply reporting using tools with default settings. Our review has revealed heterogeneity in the number of tasks, the order of these within pipelines, and the tools used to implement them, along with a lack of even basic adaptations to the COI metabarcode for most of the papers. The majority of available software and resources for metabarcoding bioinformatics are still those that have been developed around the 16S rRNA gene (the primary target for microbiome metabarcoding), including the most popular software packages (e.g., USEARCH) and sets of wrapper scripts (e.g., QIIME, OBItools). While in many cases these methods may carry over to COI without issue, we observe very few studies that report consideration or analysis that assesses or validates the suitability of software choices for COI. These issues suggest that the expansion of wocDNA COI metabarcoding is proceeding at a pace and manner that could lose sight of or simply ignore the challenges inherent in producing high‐quality data and reproducible methods (Baker et al., 2016; Zinger et al., 2019), and lose out on the potential for exploiting the benefits of the COI marker for wocDNA metabarcoding of Metazoa.

DNA metabarcoding has broad multidisciplinary potential, as demonstrated by the expansion in use of metazoan wocDNA COI metabarcoding among users from very diverse backgrounds. The diversity of applications of metabarcoding requires the concomitant bioinformatic techniques to be flexible and adaptable, and the field remains under active development. Thus it would not be productive to attempt to prescribe pipelines, tasks or even software tools in the name of standardisation, as there is no one‐size‐fits‐all approach in metabarcoding. However, some degree of harmonisation is required to ensure quality, reproducibility and potential integration in metastudies (Tedersoo et al., 2015). Additionally, the absence of a harmonised framework of bioinformatic processing can act as a barrier for potential new users (Liu et al., 2020), hampering the growth of the field. To these ends, we thus propose a set of recommendations that we believe all researchers in the field should consider when designing and reporting their wocDNA COI metabarcoding bioinformatics pipeline, with the hope that they will catalyse harmonised implementation.

**Fully report all tasks, software, software versions and parameters used, even if just the defaults.** Our results show that underreporting is a recurrent problem. Comprehensive reporting of the tasks, pipelines and software used is essential for further integrating results in future reviews or meta‐analyses (Tedersoo et al., 2015). Furthermore, care should be taken to report not just the name of the software package, but also the exact function, and if wrapper scripts are used then the underlying functions should be reported. Considering the trade‐off with current constrictions for manuscript length, this could be achieved by the inclusion of a supporting table following the STAR‐METHODs philosophy (Marcus, 2016), where task reference, order within the pipeline and software used are included. Note that the task lexicon and software lists compiled in this review (see Table 1) are a very useful resource for this purpose. This reporting effort for all the wocDNA COI metabarcoding will promote rigour and robustness with an intuitive, consistent framework that makes reporting easier for the author and replication easier for the reader.

**Implement filtering tasks such that spurious sequences are sufficiently removed to meet the assumptions of the research question.** The quality of metabarcoding results is likely to depend most on the appropriate inclusion of filtering into a pipeline (Calderón‐Sanou et al., 2020; Elbrecht et al., 2018; Zinger et al., 2019), so proper implementation of filtering tasks are critical for robust and harmonised use of COI metabarcoding. In metabarcoding, real amplicon sequence variants (ASVs, Callahan et al., 2017) amplified from target genes are inherently accompanied by spurious sequences, arising from multiple sources. Indeed, taxonomic inflation is a recurring issue demonstrated in communities with known haplotype composition (Creedy et al., 2020; Elbrecht et al., 2018). This can be exacerbated for mitochondrial markers like COI, due to the coamplification of NUMTs and other nonauthentic ASVs that are missed by denoising and require stringent, optimised filtering based on read abundances such as that implemented by the metaMATE software (Andújar et al., 2021). To ensure quality and reproducibility, metabarcoding studies should consider implementing the six most common filtering approaches, that is, quality, length, Chimera, translation, and frequency filtering, plus denoising. For each of these tasks, appropriate thresholds should be considered, implemented and fully reported to a level that ensures reproducibility (see e.g., Antich et al., 2021). Given the demonstrated importance of these tasks for most wocDNA metabarcoding studies, if any are not employed by a study the omission should be explained.

**Adapt pipelines to the COI fragment.** Suitable adaptations include read processing and filtering steps that leverage evolutionary properties of the protein coding nature of this fragment, or determining appropriate parameters for tools originally designed for other DNA regions. Some recent advances have been made in filtering tasks (metaMATE, Andújar et al., 2021; coil, Nugent et al., 2020; entropy‐based denoising, Turon et al., 2020; Antich et al., 2021) but further development in these promising areas is essential to fully exploit the potential of the COI gene for metabarcoding. As mentioned previously, there are no tools that enable simple length filtering variation that accounts for codon‐level insertion or deletion. To our knowledge there is limited work exploring the extent to which protein structure inference might allow identification of erroneous sequences: for example the SOAPbarcode pipeline (Liu et al., 2013) includes a script that filters sequences based on translation hydrophilicity, but this is not comprehensively documented or discussed in the associated publications. Computation of protein structural properties is relatively trivial to perform, and seems like a fertile ground for novel development of filtering tools for protein coding markers.

**For each task, consider all software available and try to select the most appropriate tool(s).** This can only be approached with sufficient information about available software, and to this end we include a list of all software used for each task within Table S1, and Table S3 includes links to documentation and publications. The selection of the most appropriate tool is not always straightforward, but we suggest considering (i) the extent to which the tool was designed for the intended barcode region, purpose or data set, (ii) the detail of available documentation and explanation to ensure a tool performs as expected, (iii) the availability and flexibility of options to appropriately apply the tool, (iv) the frequency of use of a tool in other studies with similar research aims, and (v) all else being equal, the simplest approach. Ideally, where multiple approaches exist, reasonable comparison between key methods should take place to fully understand the potential variation in conclusions that might arise from different bioinformatic choices, and the results of these comparisons should be reported. This is particularly the case when considering alternative, conceptually distinct algorithms for more bioinformatically complex tasks, such as denoising and OTU delimitation. The development of software packages and open access platforms integrating a catalogue of common bioinformatic tools, such mBRAVE (http://www.mbrave.net/), may play a fundamental role towards a proper selection and harmonisation of the software used. However, software choices should be made on the basis of appropriateness and usefulness, rather than simply ease of availability and implementation due to inclusion in these packages/platforms. Choice of software tools and pipeline design should be careful not to be biased by tools and pipelines designed for nonmitochondrial loci (Antich et al., 2021).

**Verify the compatibility of the tasks within a pipeline, especially with respect to task order.** It is important to ensure that the assumptions of one task have not been violated by upstream processing; for example, UNOISE denoising employs a model of error rates in Illumina sequencing, and if errors have been removed by prior length or frequency filtering this model may not accurately fit to the data. Further, linked processes should be compatible: for instance, if OTU delimitation is based on a linkage algorithm such as swarm (Mahé et al., 2015), it is inappropriate to employ a simple similarity‐based mapping method to assign reads to the resultant OTUs.

Aside from these recommendations, we also urge researchers to make data publicly available, both raw reads and final ASV and/or OTU sequences. Raw read data sets will become an invaluable resource for future work integrating many wocDNA metabarcoding studies across spatial and temporal scales, with continuing development and improvement of bioinformatic pipelines allowing for forward‐compatibility of the data as analytical tools continue to evolve. Uploading ASV and/or OTU sequences, even with incomplete taxonomy, improves the capability of methods for taxonomic assignment that draw on these resources and provides fertile data sets for future development of bioinformatic methods.

## 4 CONCLUSIONS

The past decade has seen rapid growth in the development, testing and use of wocDNA COI metabarcoding. Much effort has been expended in the development of laboratory, sequencing and bioinformatic methodologies for wocDNA COI metabarcoding and for metabarcoding as a whole. However, while much progress has been made towards harmonisation of lab and sequencing methods, bioinformatic processes have remained a tangle of varying software, pipelines and theoretical approaches, often suffering from underreported detail. This diversity allows for versatility, especially for those who are well informed and experienced in bioinformatics and able to pick and choose the appropriate approach. However, choosing from the range of approaches could easily hinder new applications of metabarcoding for researchers coming from a limited bioinformatic background, and high heterogeneity can stymie the potential for future reviews and meta‐analyses. Our review, which is the first evaluating the state of the art on this topic, highlights that this danger is clearly present in the field of metazoan wocDNA COI metabarcoding. The results of our assessment and the recommendations derived from it may help to improve bioinformatic harmonisation and thus the long‐term integrative potential of wocDNA COI metabarcoding for biodiversity science.

## CONFLICT OF INTEREST

Alfried P. Vogler is a cofounder and scientific advisor of NatureMetrics, a private company providing commercial services in DNA‐based monitoring. The authors declare that they have no other conflicts of interest.

## AUTHOR CONTRIBUTION

Thomas J. Creedy and Paula Arribas conceived the study. Thomas J. Creedy and Paula Arribas assessed the initial paper set for inclusion, Thomas J. Creedy evaluated the methods of the core paper set and analysed the data. Thomas J. Creedy and Paula Arribas wrote the initial draft and all coauthors contributed to the final manuscript.

## Supporting information

App S1

Tab S1‐S3

## Data Availability

Appendix S1 (methods, figures and tables) give the full details and methodological evaluation of the 111 publications making up the core papers.
