# Supplementary material for: Coming of age for COI metabarcoding of whole organism community DNA: Towards bioinformatic harmonisation
Source: Mol Ecol Resour. 2021 Sep 30;22(3):847–61. doi: 10.1111/1755-0998.13502 (PMC9292290; doi:10.1111/1755-0998.13502)
Supplement: Supplementary file 1 — App S1 [file MEN-22-847-s001.docx]

**Appendix S1**

**SUPPLEMENTARY MATERIAL**

**Coming of age for COI metabarcoding of whole organism community DNA: towards bioinformatic harmonisation**

Creedy T.J.^1*^, Andújar C.^2^, Meramveliotakis E.^3^, Noguerales V.^2,3^, Overcast I. ^4^, Papadopoulou A.^3^, Morlon H.^4^, Vogler A.P.^1,5^, Emerson B.C.^2^ & Arribas P.^2^

^1^ Department of Life Sciences, Natural History Museum, Cromwell Road, London SW5 7BD

^2^ Instituto de Productos Naturales y Agrobiología (IPNA-CSIC), 38206, S.C. La Laguna, Spain

^3^ Department of Biological Sciences, University of Cyprus, Nicosia, Cyprus

^4^ Institut de Biologie de l’ENS (IBENS), Département de biologie, École normale supérieure, CNRS, INSERM, Université PSL, Paris, France

^5^ Department of Life Sciences, Imperial College London Silwood Park Campus, Ascot

* Corresponding author: thomas@tjcreedy.co.uk

**Supplementary Methods1**

The 111 selected papers were systematically processed to record the nature of the study presented and the bioinformatics procedures applied. As well as standard publication information (***journal***, ***year***), we classified each article as follows:

***Research aim,*** one of:

**methodological**, where the primary aim is to study aspects of metabarcoding methodology including primer design or empirical analysis of differential recovery rates under varying lab or bioinformatic procedures

**pilot**, where the primary stated aim is a proof-of-concept or feasibility study into the success of metabarcoding for uncovering accurate community data in the taxon/community/biome studied.

**empirical**, where the primary aim and results concentrate on empirical analysis of ecological patterns uncovered by use of metabarcoding.

***Material used***, one of:

**real**, where actual community samples, collect from some sort of bulk or batch sampling, were metabarcoded. This includes studies where the bulk sample was subsequently subsampled, modified or reconstructed as part of the study.

**artificial**, where the metabarcoded community composition was constructed from individually-selected specimens.

***Sample modification***, one of:

**none**, where community samples were not modified in any of the following ways

**size normalisation**, where the DNA contribution from different size fractions of the community were standardised by tissue subsampling and/or proportional combination of separate DNA extracts

**taxonomic separation**, where a bulk sample was sorted by taxon and taxonomic subsets received different treatment (e.g. varied extraction methods, differential DNA quantity inclusion in final pool, separate metabarcoding, exclusion)

**constructed**, where a bulk sample was substantially re-configured for the aims of the study in terms of composition such that it no longer reflected the original community composition (if relevant). All “artificial” material were part of this category.

**pooled**, where multiple bulk samples were pooled into fewer bulk samples with the effect of discarding fine compositional resolution

***Pipeline comparison***, whether or not the article presented and contrasted the use of alternative pipelines, software or parameters at any stage of bioinformatic processing. To be counted, the paper must compare the alternatives as mutually exclusive, for example two different OTU delimitation methods, not synthesise the results from multiple alternatives, for example using multiple taxonomic assignment methods and picking the best results for each OTU.

Whether or not the article completely reported the:

***Name*** of software used for every step of the pipeline

***Version*** of each piece of software used

The ***parameters*** used for each piece of software used (or a statement that defaults were used)

Whether or not the reader’s most complete possible understanding of the pipeline used relied on reading the methods section of a separate publication, i.e. the authors of a paper did not fully outline the methods and simply cited another article.

Subsequently, the methods section of each paper was systematically parsed to understand the bioinformatics procedures used. For this purpose we define a set of standard terms.

A **task** is a specific, self-contained action in a pipeline, generally with a clearly-defined purpose and performed by a single tool.

A **tool** is a specific piece of software, and in many cases a function within that software.

A **pipeline** is the specific sequence of steps in a specific order, each comprising a tool used to carry out a particular tasks

For example, the fifth step in a pipeline may comprise the task of OTU delimitation, carried out by the cluster_otus function in the USEARCH software, which together comprise a specific tool. An alternative tool for this task is the Swarm software, which only performs OTU delimitation so no reference to a particular function is needed.

The pipeline used by each article was identified as far as possible based on text, figures, supplementary material and/or cited papers. For each step in the pipeline, the numerical order, software, function and task were recorded. Where the software used was not reported, “NR” was recorded. If a specific function of the named software was reported this was recorded, otherwise this was left blank. In some cases, authors reported only a function as the tool used, and not the parent software suite - in these cases, for consistency across the database, we added the software to our database as if it were stated in the paper. However, in the inverse case, where the reported tool was only a software suite and the specific function within this was not reported, we did not add the function. For example, see table X; USEARCH has multiple OTU delimitation methods and it can’t be known which was actually used in this case. Note that in this example we would also have recorded this article as failing to fully report the software used (see above).

| **Reported Method** | **Recorded data** | | |
| --- | --- | --- | --- |
|  | **Software** | **Function** | **Task** |
| “We used cluster_otus to pick OTUs” | USEARCH | cluster_otus | OTU delimitation |
| “We used USEARCH to pick OTUs” | USEARCH |  | OTU delimitation |

In cases where it was stated or implied that no automated tool was used, and instead the authors manually inspected and processed sequence data, “MANUAL” was recorded despite this likely happening within some multi-function GUI software package (such as Geneious). However, in some cases authors stated they used such a GUI software package to a to perform a specific task, but did not state what function within this package was used. In this case, we recorded the name of the software, as we assumed it was more likely an automated function was used rather than manual processing.

Some software packages utilise each other’s tools, for example the QIIME package utilises functions from USEARCH as well as other OTU delimitation tools. These are often run within wrapper scripts, often with a different name to that of the true software or function called. As we could not be confident that we would be able to always identify tools as wrappers, we chose to record the tool as stated in the paper, not the underlying tool, unless the authors clearly and unequivocally reported the underlying tool and that the overlying tool was simply a wrapper or pointer.

Finally, for each step the task was recorded if this was clear, or could be unequivocally determined from the tool used, otherwise “NR” was recorded. Where a single piece of software was stated to be used to perform multiple clearly separable tasks, this was recorded as multiple steps using the same software. Where the order of steps was not unequivocal, we used the order in which they were mentioned in the text. In the relatively rare case that non-mutually-exclusive steps were performed in parallel (generally only taxonomic assignment by multiple different tools that was later synthesised), these were recorded in the order in which they were stated in the text. Where multiple mutually exclusive steps or pipelines were employed for the purposes of comparison of pipelines, we recorded that pipeline that the authors concluded to be empirically superior, or from which the authors used the output data for subsequent analysis.

We focused solely on steps that were a part of metabarcoding and that were bioinformatic in nature, i.e. primarily dealt with DNA sequence data for the purposes of generating OTU information. Our recorded data thus ends with steps that convert sequence data into data comparable to that of traditional ecological studies, i.e. sample by OTU tables and taxonomic identifications of OTUs. We did not record any steps performing phylogenetics with a final OTU set, although we recorded steps where phylogeny-based methods were used as part of OTU delimitation and filtering. Similarly, we did not record normalisation or other analytical pre-processing steps performed on the final sample by OTU table, but we did record normalisation when this was performed prior to OTU delimitation.

As papers were processed, we automatically added to a database of software, functions and task names used in order to keep our classifications consistent despite varying terms used, particularly for tasks. We initially determined a set of around 10-15 task categories, but this expanded as we identified previously unconsidered tasks and in a small number of cases split categories into more detailed terms. For each entry in the software database, we recorded the following:

Whether or not the entry was for a standalone application or a software package - the latter comprising multiple bioinformatic functions in a single application (e.g. Geneious, USEARCH) or linked sets of functions under a unifying umbrella (e.g. QIIME, OBItools)

Whether the entry was a command-line tool (CLI) or was principally operated through a graphical user interface (GUI)

The principal programming language the tool was written in

Whether the software is principally a standalone installable program, a script, a library designed to be used within a specific programming language or available only on a webserver.

The license available for academic use, one of Free - all functionality indefinitely available for no charge; Mixed - many functions indefinitely available for no charge, but some functions unlocked only by payment; Proprietary - the majority of functions indefinitely available only through purchase.

The year the software was last updated, as far as could be ascertained

The source publication and most direct URL for access to the software

**Supplementary Figures**


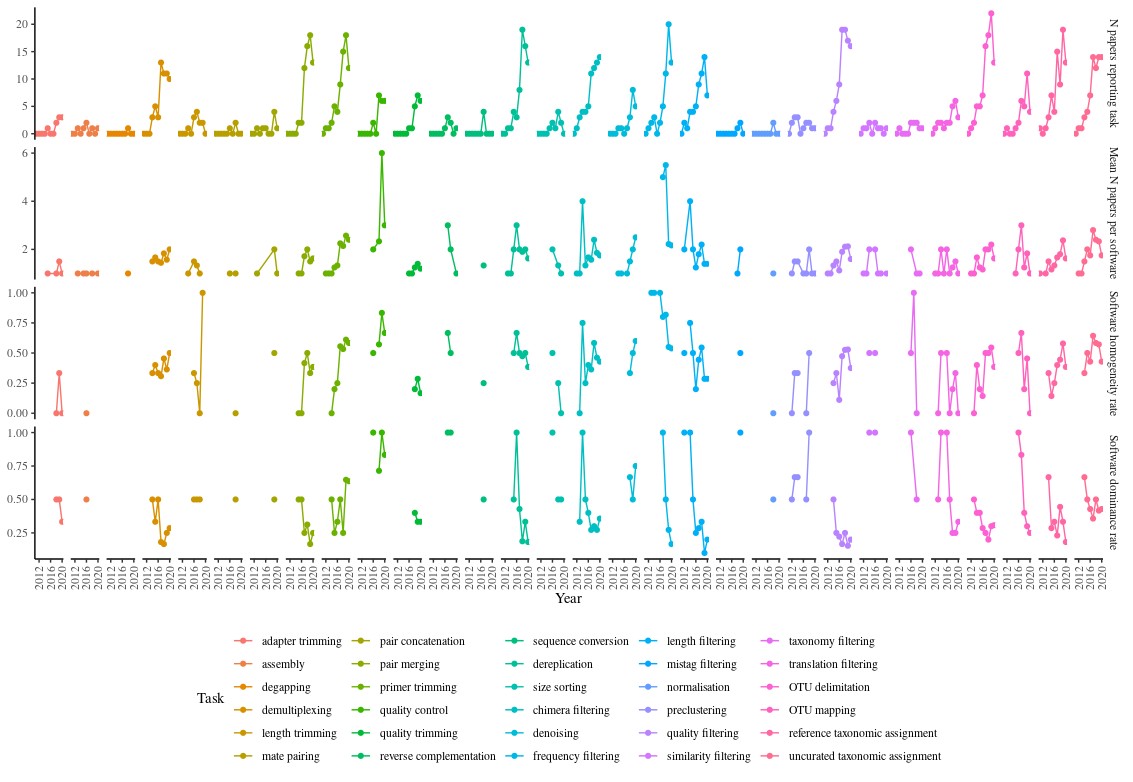


**Figure S1:** *Task and software use over time. Panels show (left to right) different metabarcoding bioinformatic tasks employed, and (top to bottom) the proportion of papers published in a given year that report the specified task, the mean number of papers published in a given year per software tool used in that year, the software homogeneity rate and the software dominance rate (see description in Figure 5)*

**Supplementary Tables [available in supplementarytables.xslx]**

**Table S1** [sheet 1: SupplementaryTable1_MT1-extended]: *Table of all bioinformatic tasks performed across the core papers set. Tasks are grouped into four groups by broad purposes, and a detailed definition of each task is given along with summary statistics of the implementation of each task across the 111 papers. The software functions column lists the software, software packages and/or software package and subsidiary functions reported to have been used for each task, along with the number of papers reporting each use in parentheses. Note that some papers employ the same task multiple times, either with different parameters, software or positions in the pipeline, hence the sum of software function incidence may be greater than the number of papers reporting a task.*

**Table S2** [sheet 2: SupplementaryTable2_papersdata]: *The bioinformatic pipelines performed by the 111 selected papers. For explanations of the columns, see methods and Supplementary methods*

**Table S3** [sheet 3: SupplementaryTable3_software]: *All software recorded from the bioinformatic methods used by the 111 selected papers. For explanations of the columns, see Supplementary methods*
